# ATF3 and JDP2 deficiency in cancer associated fibroblasts promotes tumor growth via SDF-1 transcription

**DOI:** 10.1038/s41388-019-0692-y

**Published:** 2019-01-22

**Authors:** Shimrit Avraham, Ben Korin, Sharon Aviram, Dvir Shechter, Yuval Shaked, Ami Aronheim

**Affiliations:** 1 Department of Cell Biology and Cancer Science, The B. Rappaport Faculty of Medicine, Technion - Israel Institute of Technology, Haifa, Israel; 2 Department of Immunology, Department of Neuroscience, The B. Rappaport Faculty of Medicine, Technion - Israel Institute of Technology, Haifa, Israel

**Keywords:** Cancer genetics, Cancer microenvironment

## Abstract

The activating transcription factor 3 (ATF3) and the c-Jun dimerization protein 2 (JDP2) are members of the basic leucine zipper (bZIP) family of transcription factors. These proteins share a high degree of homology and both can activate or repress transcription. Deficiency of either one of them in the non-cancer host cells was shown to reduce metastases. As ATF3 and JDP2 compensate each other’s function, we studied the double deficiency of ATF3 and JDP2 in the stromal tumor microenvironment. Here, we show that mice with ATF3 and JDP2 double deficiency (designated thereafter dKO) developed larger tumors with high vascular perfusion and increased cell proliferation rate compared to wild type (WT) mice. We further identify that the underlying mechanism involves tumor associated fibroblasts which secrete high levels of stromal cell-derived factor 1 (SDF-1) in dKO fibroblasts. SDF-1 depletion in dKO fibroblasts dampened tumor growth and blood vessel perfusion. Furthermore, ATF3 and JDP2 were found to regulate SDF-1 transcription and secretion in fibroblasts, a phenomenon that is potentiated in the presence of cancer cells. Collectively, our results suggest that ATF3 and JDP2 regulate the expression of essential tumor promoting factors expressed by fibroblasts within the tumor microenvironment, and thus restrain tumor growth.

## Introduction

The tumor microenvironment (TME) is a key element supporting tumor growth and proliferation through the interaction between cancer and stromal cells. Stromal cells are divided into three main classes: infiltrating bone marrow derived cells (BMDCs), angiogenic vasculature endothelial cells and cancer associated fibroblasts (CAFs) [1]. Intracellular communication between the different cell types in the stroma occurs through the secretion of numerous cytokines, chemokines and growth factors that are known to regulate cancer cell fate [2]. For example, endothelial cells secrete growth-promoting factors such as VEGF, eNOS and angiotensin II, independent of blood-borne factors that support the tumor vasculature, a process known as angiogenesis [3, 4]. Similarly, CAFs secrete signaling proteins such as EGF and IGF-1 which promote cancer cell proliferation [5–7]. Therefore, studying the role of the stroma in tumor growth can provide a greater understanding of tumor development and lead to the development of novel treatment strategies to block it.

Activating transcription 3 (ATF3) and c-Jun dimerization protein 2 (JDP2) are members of the basic leucine zipper (bZIP) protein superfamily of transcription factors [8–10]. Both proteins share a high degree of homology within the bZIP domain and display multiple similar functions. ATF3 and JDP2 repress transcription and designated hereafter as bZIP repressors. Nevertheless, both can activate transcription depending on the protein partner [11] and promoter context [9, 12].

While JDP2 is constitutively expressed in all cells tested [13, 14], ATF3 is an immediate early gene, highly induced in response to multiple cell stresses [15]. The bZIP repressors can bind as homo or hetero-dimers to DNA sequences such as TPA response elements (TRE) and cyclic adenosine mono phosphate response elements (CRE). Upon dimerization, the bZIP repressors inhibit transcription of TRE and CRE containing promoters. Transcription inhibition occurs by recruitment of multiple histone deacetylase (HDAC) to TRE and CRE containing promoters [14, 16].

Various studies suggest that ATF3 and JDP2 have a dual role in malignant transformation. ATF3 suppresses cell cycle progression in HeLa cells and Ras-stimulated tumorigenesis [17, 18], yet is elevated in breast tumor samples [19]. Similarly, JDP2 prevents AP-1 transcription interfering with the oncogenic properties of c-Jun [20], but in JDP2 transgenic mice, JDP2 expression in the liver promotes hepatocellular carcinoma [21]. In humans, high expression of ATF3 or JDP2 in the tumors of lung cancer patients correlates with poor survival [22] (Fig. S1). In host cells within the tumor microenvironment, it was recently demonstrated that mice lacking ATF3 (ATF3-KO) or JDP2 (JDP2-KO) display a reduced rate of metastasis in polyoma middle T-antigen (PyMT) breast carcinoma mouse model and/or Lewis Lung Carcinoma (LLC) [23, 24]. Since JDP2 and ATF3 may compensate for one another in the absence of their counterpart [25], we examined the effect of double deficiency of ATF3 and JDP2 (dKO) on tumor growth and metastasis. We found that while a single deficiency of either ATF3 or JDP2 resulted in lower metastasis [23, 24], there was no change in pulmonary metastasis burden between dKO and WT mice in the experimental set-up of our study. In our experimental model, we found that tumor growth was significantly higher in the dKO mice than WT mice, indicating that ATF3 and JDP2 expression by host cells contribute to the primary tumor fate. Therefore, here we describe the molecular consequence of bZIP repressors deficiency in stromal cells on tumor growth. We identified that the deficiency of ATF3 and JDP2 elevates stromal cell-derived factor 1 (SDF-1) secretion by CAFs found in the tumor stroma, ultimately increasing tumor growth. Taken together, our results highlight the importance of studying the role of host transcription factors upon tumor development, and may provide possible therapeutic venues for cancer treatment.

## Results

### dKO mice exhibit increased tumor growth and cancer cell proliferation

To investigate the impact of host JDP2 and ATF3 expression on tumor growth and proliferation, we generated mice with double deficiency of these proteins by consecutive crossing of JDP2^−/−^ and ATF3^−/−^ strains. The mice were born in a Mendelian distribution and do not display an overt phenotype.

**Fig. 1:**
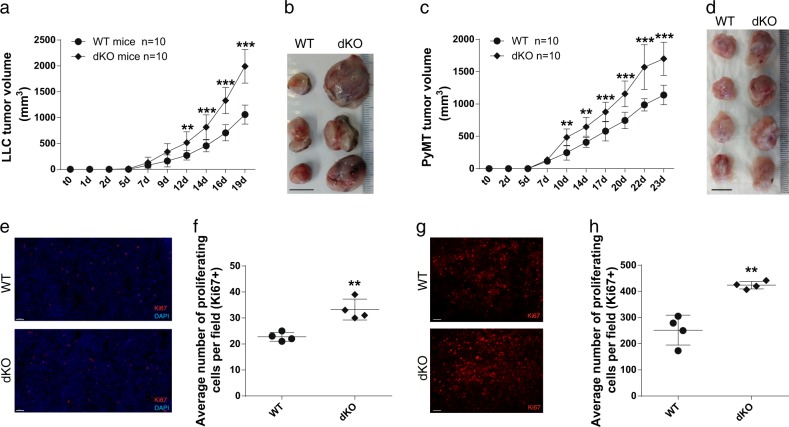
ATF3 and JDP2 double deficiency enhances tumor growth and proliferation. **a** WT and dKO mice were subcutaneously implanted into the flanks with LLC cells (0.5 × 10^6^ cells per mouse). Tumor volume was monitored over time using the formula: width^2^ × length × 0.5. Data are presented as mean ± SD, Two-way repeated-measures ANOVA followed by Bonferroni post-tests, *n* = 10, **p < 0.01; ***p < 0.001. Representative image of at least three experiments. **b** Representative image of tumors derived from **a**. **c** WT and dKO mice were orthotopically implanted into the mammary fat pad with PyMT cells (1 × 10^5^ cells per mouse). Tumor volume was monitored over time and analyzed as described in **a**. Data are presented as mean ± SD, Two-way repeated-measures ANOVA followed by Bonferroni post-tests, *n* = 10, **p < 0.01; ***p < 0.001. Representative image of at least three experiments. **d** Representative image of tumors derived from **c**. **e**–**h** Representative images of tumor sections from **a** (**e**) and **c** (**g**) immunostained for Ki67 (red) and DAPI (blue). Scale bar, 50 µm and 100 µm, respectively. **f**, **h** Quantification of the number of proliferating cells (Ki67+) of tumor sections in LLC (**f**) and PyMT (**h**). Each dot represents the mean of 5 fields taken from one mouse. Data are presented as mean ± SD, Student’s *t*-test, *n* = 4, **p < 0.01. Representative image of at least three experiments

WT and dKO mice were subcutaneously implanted into the flank with LLC cells. Tumor growth was monitored over time and mice were sacrificed when the primary tumors reached endpoint in one of the groups. Lungs were dissected and sections were stained for micro-metastasis using H&E staining. At the experiment endpoint, we did not detect any significant difference in metastatic lesions in the lungs of WT and dKO mice (Fig. S2a, b). In contrast, tumors of dKO mice were 2–3 times larger than those of WT mice (Figs. 1a, b). Using a breast cancer model, in which polyoma middle T cells (PyMT) were implanted into the mammary fat pad of female mice, we similarly detected no change in metastases lesion (Fig. S2a, c). However, we detected larger tumors in dKO mice when compared to WT mice (Fig. 1c, d). To ensure that the increase in tumor growth is specific to dKO mice, we repeated LLC cell implantation in the flank of single knockout mice. While tumor size in ATF3-KO mice was similar to WT mice, the tumors developed in JDP2-KO mice were significantly larger (Fig. S3a, b), consistent with previous publications [23, 24]. Importantly, tumors developed in dKO mice were significantly larger compared to tumors grown in JDP2-KO mice (Fig. S3a, b).

Next, we examined whether the increase in tumor size in dKO mice is due to tumor cell proliferation or stromal cell recruitment. We first stained LLC and PyMT tumor sections for Ki67 (a cell proliferation marker). In accordance with the increase in tumor size, tumor sections derived from both LLC and PyMT tumors of dKO mice displayed a significantly higher number of proliferating cells per field than tumors developed in WT mice (Fig. 1e–h). Thus, in both tumor models, dKO mice exhibited larger tumor size due to increased rate of cell proliferation compared to those developed in WT mice.

### ATF3 and JDP2 deficiency in the stromal tumor microenvironment enhances blood vessel perfusion

To examine which of the stromal TME cellular components affects tumor growth, we focused on the three most abundant stromal cell populations of the tumor: infiltrating immune cells, endothelial cells and CAFs. To test the effects of infiltrating immune cells, we performed a bone marrow (BM) transplantation experiment in which BM cells, derived from either dKO or WT mice were injected intravenously (i.v.) to lethally irradiated WT mice. Transplantation efficiency was validated by PCR after 8 weeks (not shown). Then, we implanted LLC cells into the chimeric mice and assessed tumor growth. We did not detect any significant differences in LLC tumor growth between chimeric mice transplanted with either WT or dKO BM (Fig. S4). These results indicate that BM derived immune cells of dKO mice do not affect tumor growth.

**Fig. 2:**
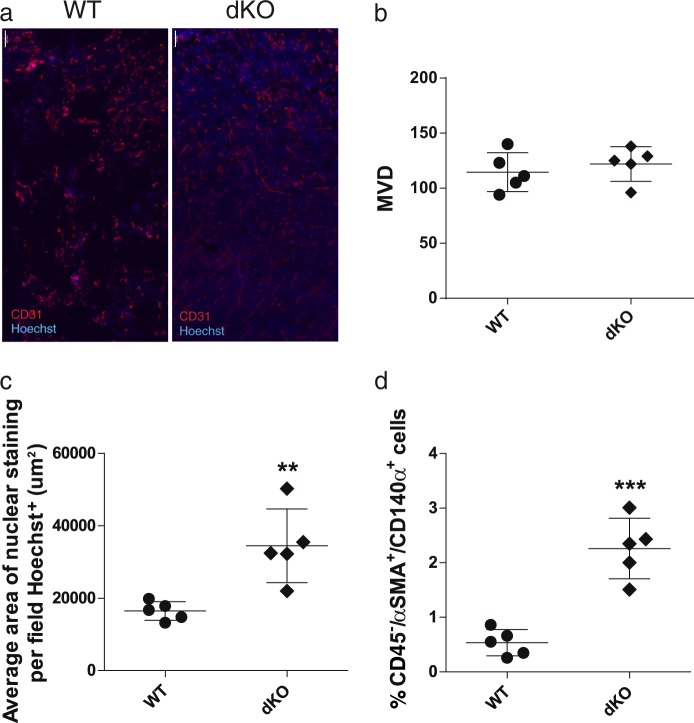
Tumors developed in dKO mice display higher blood vessel perfusion and fibroblasts recruitment. **a** Representative images of LLC tumor sections derived from WT and dKO mice, stained with anti-CD31 (endothelial cells; red) and Hoechst (nuclei; blue). Scale bar, 100 µm. **b**, **c** Quantification of micro vessel density (MVD, **b**) and nuclear staining area representing perfusion (**c**) per field. Each dot represents the mean of 5 fields taken from one mouse. Data are presented as mean ± SD, *n* = 5, Student’s *t*-test, **p < 0.01. **d** Single cell suspensions of tumors derived from WT or dKO mice immunostained for fibroblasts (CD45^neg^/αSMA^+^/CD140α^+^) and analyzed by flow cytometry. Each dot represents one mouse. Data are presented as mean ± SD, Student’s *t*-test, *n* = 5, ***p < 0.001. Representative images of at least three experiments

We then examined the tumor endothelial cells, known as important contributors to tumor growth [26]. We assessed micro-vessel density (MVD) by staining tumor sections for CD31 (endothelial cell marker), and characterized blood vessel perfusion using i.v. injection of Hoechst reagent one minute prior to sacrifice. Our analysis revealed no significant difference in the number of micro-vessels per field in tumor sections derived from both WT and dKO mice (Fig. 2a, b). However, blood vessels in tumors derived from dKO mice were more perfused compared to blood vessels in tumors of WT mice (Fig. 2a, c). We detected similar results in both parameters in PyMT derived tumors (Fig. S5a-c).

Lastly, we examined the possible contribution of CAFs to tumor growth in dKO versus WT mice. We extracted LLC tumors developed in WT and dKO mice, and dissociated them into a single cell suspension for flow cytometry analysis. CAFs were defined as CD45^negative^/αSMA^+^/CD140α^+^, as previously described [5]. In our analysis, tumors derived from dKO mice had a significant increase in the percentage of CAFs of total single cells, compared to those of WT mice (Fig. 2d).

Collectively, these results indicate that the double deficiency in ATF3 and JDP2 specifically affects fibroblasts in the tumor environment and the function of tumor vasculature, possibly leading to changes in tumor development and size.

### ATF3 and JDP2 deficiency in fibroblasts promotes tumor growth and blood vessel perfusion

**Fig. 3:**
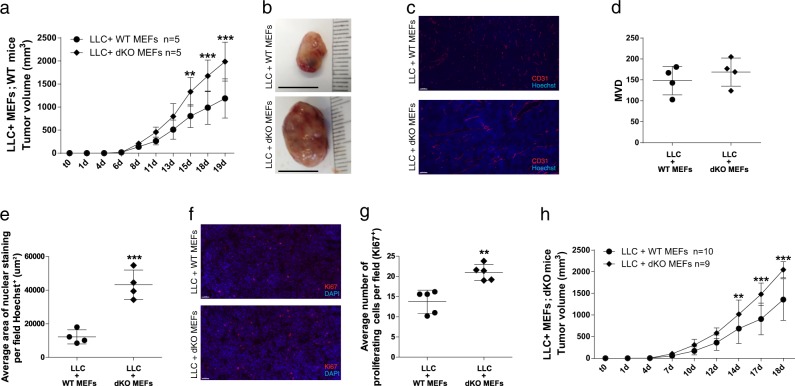
dKO MEFs promote LLC tumor growth, cell proliferation and blood vessel perfusion. **a** WT mice were subcutaneously implanted into the flanks with Matrigel containing cell mixture composed of LLC cells (0.5 × 10^6^ cells per mouse) together with MEFs (1.5 × 10^6^ cells per mouse) derived from either WT or dKO mice. Data are presented as mean ± SD, Two-way repeated-measures ANOVA followed by Bonferroni post-tests, *n* = 5, **p < 0.01; ***p < 0.001. Representative image of at least three experiments. **b** Representative images of tumors from **a**. **c** Representative images of tumor sections from **a**, stained with anti-CD31 (endothelial cells; red) and with Hoechst (nuclei; blue). Scale bar, 100 µm. **d**, **e** Quantifications of microvessel density (MVD, **d**) and nuclear staining (**e**) per field. Each dot represents the mean of 5 fields taken from one mouse. Data are presented as mean ± SD, Student’s *t*-test, *n* = 4, ***p < 0.001. **f** Representative images of tumor sections from **a**, stained with anti-Ki67 and DAPI. Scale bar, 50 µm. **g** The number of proliferating cells (Ki67^+^) per field. Each dot represents the mean of 5 fields taken from one mouse. Data are presented as mean ± SD, Student’s *t*-test, *n* = 5, **p < 0.01. Representative images of at least three experiments. **h** dKO mice were subcutaneously implanted as described in **a**, and tumor volume was monitored over time. Data are presented as mean ± SD, Two-way repeated-measures ANOVA with Bonferroni post-tests, *n* = 10,9, **p < 0.05; ***p < 0.001. Representative image of two experiments

To understand the possible role of fibroblast recruitment of to the tumor stroma, we subcutaneously (s.c.) implanted WT mice with Matrigel, containing a cell mixture of LLC cells (0.5 × 10^6^) and mouse embryonic fibroblasts (MEFs, 1.5 × 10^6^), from either dKO or WT embryos. In mice co-implanted with dKO MEFs, tumor size was twice the size of tumors developed in the presence of WT MEFs (Fig. 3a, b). Moreover, MEFs derived from JDP2-KO mice co-implanted with LLC cells also developed larger tumors compared to WT and ATF3-KO MEFs (Fig. S6). Nonetheless, these tumors were significantly smaller as compared to those with dKO MEFs (Fig. S6). We identified similar results when the same experimental design was performed with PyMT cells (Fig. S7). Next, we asked whether the mechanism of this MEF-related effect relies on the two main processes we identified before: cell proliferation and blood vessel perfusion. We stained the tumor sections in each group for Ki67, CD31 and Hoechst, and while MVD was similar (Fig. 3c, d), the blood vessels in the tumors co-implanted with dKO MEFs displayed higher perfusion, compared to tumors co-implanted with WT MEFs (Fig. 3c, e). In addition, tumors co-implanted with dKO MEFs exhibited up to 30% more proliferating cells, compared to tumors co-implanted with WT MEFs (Fig. 3f, g). Next, we performed an inverse experiment, in which LLC or PyMT cells were co-implanted together with either WT or dKO MEFs in dKO mice. The tumors developed with co-implanted dKO MEFs were larger compared to tumors grown together with WT MEFs (Fig. 3h, Fig. S8), suggesting that the co-implanted dKO fibroblasts contributed to this process. Thus, fibroblasts lacking JDP2 and ATF3 expression, promote tumor growth and blood vessel perfusion.

### SDF-1 expression in fibroblasts is under the control of ATF3 and JDP2

**Fig. 4:**
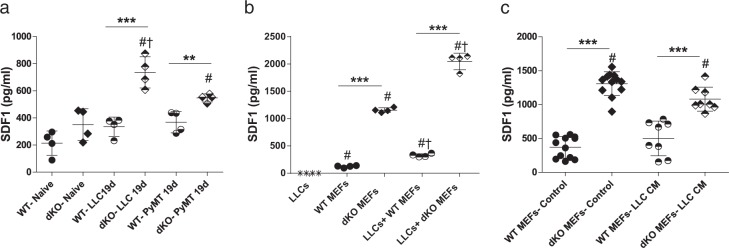
SDF-1 secretion is elevated in the presence of cancer cells. **a** Circulating serum levels of SDF-1 (pg/ml) from either naïve or LLC/PyMT tumor bearing WT or dKO mice. Each dot represents one mouse. Data are presented as mean ± SD, One-way ANOVA followed by Tukey’s post-tests, *n* = 4, *—difference compared to WT **p < 0.01; ***p < 0.001; #—difference compared to naïve mice; †—difference between tumor type. Representative image of at least three experiments. **b** WT or dKO MEFs (7.5 × 10^5^) were co-cultured with LLC cells (2.5 × 10^5^) and as a control: LLC, WT MEFs and dKO MEFs (1 × 10^6^) were cultured in serum-free (SF) medium for 24 h. The levels of SDF-1 secreted to the medium were measured (pg/ml). Each dot represents one plate. Data are presented as mean ± SD, One-way ANOVA followed by Tukey’s post-tests, *n* = 4; * - difference between genotypes- **p < 0.01; ***p < 0.001; #—difference between PyMT and LLCs; †—difference between treatments. Representative image of at least three experiments. **c** WT and dKO MEFs were cultured in the presence of 10% FBS (control) or LLC conditioned medium (CM). After 24 h, the medium was replaced with SF medium, which was then collected after 24 h. The levels of SDF-1 secreted to the medium was measured and analyzed as described in (**b**). Each dot represents one plate. Data are presented as mean ± SD, One-way ANOVA followed by Tukey’s post-tests *n* = 12,12,8,8; *—difference between genotypes ***p < 0.001; #—difference compared to WT MEFs- Control. Representative image of two experiments. In all graphs, levels of SDF-1 were assessed by ELISA

Since fibroblasts have been shown to contribute to tumor growth and proliferation, in part by inducing angiogenesis [27], we hypothesized that fibroblasts possibly mediate their effect on cancer cells via the secretion of fibroblast specific factors. Recent studies have demonstrated that SDF-1 secreted by fibroblasts contributes to tumor angiogenesis [28]. We therefore sought to determine whether SDF-1 plays a role in our experimental model. We thus examined the levels of SDF-1 in the serum of naïve and tumor bearing mice by enzyme-linked immunosorbent assay (ELISA). In naïve mice, no differences in SDF-1 levels were observed between WT and dKO mice. Yet, among tumor bearing mice (LLC or PyMT), SDF-1 levels were significantly elevated in the serum of the dKO group compared to serum derived from WT mice (Fig. 4a). We therefore examined whether the higher levels of SDF-1 found in the serum are secreted by CAFs. To examine this possibility, we measured SDF-1 secretion by MEFs. WT and dKO MEFs were cultured in the absence or presence of LLC cells (3:1 ratio respectively) and SDF-1 levels were evaluated in the conditioned medium (CM). We found higher levels of SDF-1 in media derived from dKO MEFs when compared to media with WT MEFs, both in the presence or absence of LLC cells (Fig. 4b). Importantly, the secretion of SDF-1 was more pronounced in MEFs co-cultured with LLC cells. Of note, LLC cells do not secret SDF-1 to the medium (Fig. 4b). Interestingly, we also found that ATF3-KO and JDP2-KO MEFs co-cultured with LLC cells secrete lower levels of SDF-1 than dKO MEFs (Fig. S9). Cumulatively, these results suggest a possible cross-talk between cancer cells and fibroblasts which may promote tumor growth via the regulation of SDF-1 secretion in fibroblasts.

We then tested whether the changes in SDF-1 secretion in the co-culture system are related to a direct contact between cancer cells and fibroblasts. To address this question, we isolated LLC CM and added it to MEF cell cultures, and then measured SDF-1 levels by ELISA. Despite the fact that dKO MEFs displayed higher SDF-1 levels as compared to WT MEFs, no further increase was observed when LLC CM was added (Fig. 4c), indicating that physical contact is required between LLC cells and MEFs to induce SDF-1 secretion by both WT and dKO MEFs. In vivo, this physical contact predominantly takes place at the recruitment site, the tumor microenvironment.

**Fig. 5:**
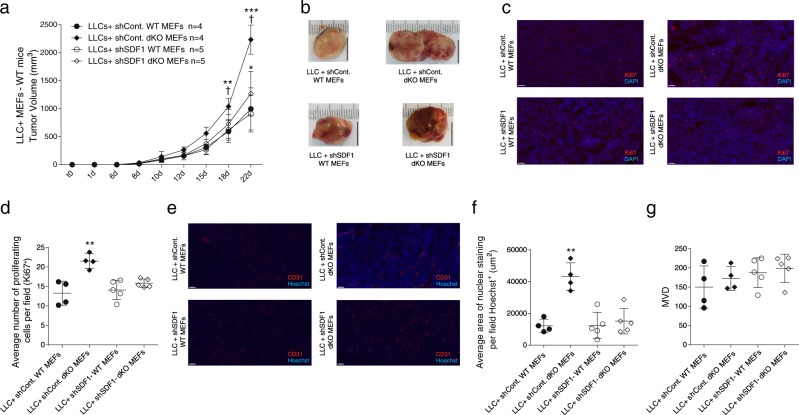
Co-implanted dKO MEFs with SDF-1 knockdown display smaller tumors and reduced rate of proliferation and blood vessel perfusion. **a** WT mice were subcutaneously co-implanted with LLC cells (0.5 × 10^6^ cells per mouse) and MEFs (1.5 × 10^6^ cells per mouse) corresponding to shControl WT and dKO MEFs or shSDF-1 WT and dKO MEFs. Tumor volume was monitored over time and analyzed as described in Fig. 1. Data are presented as mean ± SD, Two-way repeated-measures ANOVA with Bonferroni post-tests, *n* = 4,4,5,5, *—difference compared to co-implanted WT MEFs *p < 0.05; **p < 0.01; ***p < 0.001; †—difference compared to co-implanted with dKO MEFs. **b** Representative images of tumors from (**a**). **c**–**g** Representative images of tumor sections from (**a**), stained with anti-Ki67 (**c**; blue) or anti-CD31 (**e**; red) and Hoechst (nuclei; blue). Scale bar, 100 µm. Quantification of cell proliferation (**d**), perfusion (**f**), and MVD (**g**). Each dot represents one mouse. Data are presented as mean ± SD, One-way ANOVA followed by Tukey’s post-tests, *n* = 4,4,5,5, **p < 0.01; ***p < 0.001. Representative image of two experiments

Thus far, we demonstrated that higher level of SDF-1, secreted by dKO fibroblasts, is correlated with tumor growth and blood vessel perfusion. We next asked whether MEF-derived SDF-1 is necessary for tumor growth. We used shRNA directed towards SDF-1 knockdown in WT and dKO MEFs. SDF-1 shRNA transfection displayed efficient SDF-1 knockdown in MEFs, while sh-Control transfected cells were indistinguishable from non-transfected MEFs (Fig. S10). Next, the genetically modified MEFs, representing high and low SDF-1 expression from either WT or dKO mice, were co-implanted into WT mice together with LLC cells, as described above. dKO-shSDF-1 MEFs displayed a significantly reduced ability to promote LLC tumor growth (Fig. 5a, b), cell proliferative capacity (Fig. 5c, d) and blood vessel perfusion (Fig. 5e, f), when compared to dKO-sh-Control (Fig. 5a–f). Notably, no changes in MVD were observed in any of the groups tested (Fig. 5g). These results suggest that SDF-1 is necessary, at least in part, for the potentiation of the tumorigenic phenotype found in dKO mice.

### SDF-1 expression in cancer associated fibroblasts is regulated by ATF3 and JDP2

**Fig. 6:**
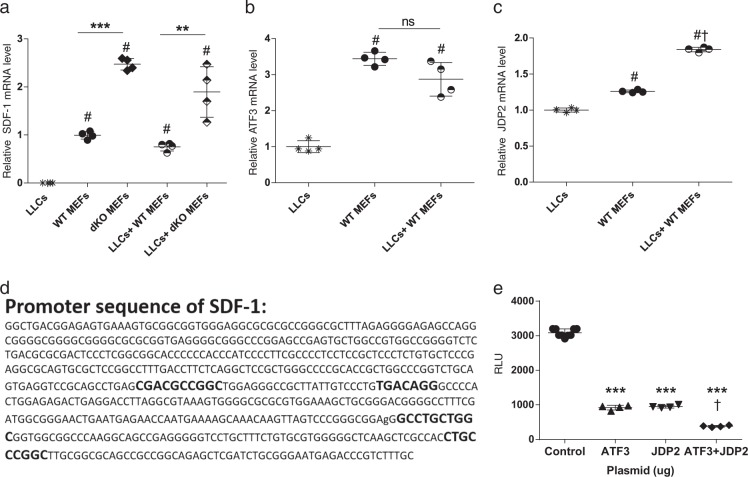
ATF3 and JDP2 suppress SDF-1 transcription. **a**–**c** WT or dKO MEFs were cultured either alone or together with LLC cells in serum free (SF) medium for 24 h. RNA was extracted and cDNA was prepared. The levels of SDF-1 (**a**), ATF3 (**b**) and JDP2 (**c**) were determined by qRT-PCR, normalized to HSP90 housekeeping gene. Data are presented as relative expression compared to WT MEF (**a**) or LLC (**b**, **c**) determined as 1. Each dot represents one plate. Data are presented as mean ± SD, One-way ANOVA followed by Tukey’s post-tests, *n* = 4; *—difference compared to WT **p < 0.01; ***p < 0.001; #—difference compared to LLCs; †—difference compared with no co-culture. Representative image of at least two experiments. **d** Sequence of the SDF-1 promoter (602 bases upstream of the transcription start site). Highlighted in bold are the putative binding sites for ATF3 and JDP2 [29]. **e** HEK 293 T cells were co-transfected with the SDF-1-luciferase reporter plasmid in the absence (empty vector) or presence of ATF3 + JDP2 expression plasmids. Luminescence was evaluated 24 h post transfection as relative light unit (RLU). Each dot represents one plate. Data are presented as mean ± SD, One-way ANOVA followed by Tukey’s post-tests, *n* = 8,4,4,4; *—difference compared to control ***p < 0.001; †—difference compared to either JDP2 or ATF3 expression plasmids. Representative image of three experiments

We further examined whether ATF3 and JDP2 expression alters SDF-1 expression at the transcriptional level. We used qRT-PCR with SDF-1 specific oligonucleotides to examine SDF-1 transcription in fibroblasts. This analysis revealed that the higher levels of SDF-1 protein in dKO MEFs compared to WT MEFs are mainly due to increased transcription in dKO mice (Fig. 6a). In contrast, the increased level of secreted SDF-1 observed following co-culture with LLC cells is likely to be mediated post-transcriptionally (Fig. 6a). The increase in SDF-1 transcription in dKO MEFs as compared to WT MEFs suggests that JDP2 and ATF3 repress SDF-1 transcription. We next examined whether JDP2 and ATF3 were expressed in MEFs using qRT-PCR. This analysis revealed that both JDP2 and ATF3 were expressed in WT MEFs at higher levels as compared to the expression in LLC cells (Figs. 6b, c). Additionally, while ATF3 levels were unchanged following co-culture with LLC, JDP2 levels increased following co-culture with LLC (Figs. 6b, c). Thus, JDP2 and ATF3 are abundant in MEFs and may regulate SDF-1 transcription.

Several putative TRE/CRE DNA binding elements within the SDF-1 promoter were previously identified (Fig. 6d) [29]. To examine whether JDP2 and ATF3 are able to regulate SDF-1 promoter, we used the SDF-1 luciferase reporter plasmid in which the SDF-1 promoter was placed upstream to the luciferase reporter gene [30]. We co-transfected the SDF-1 luciferase reporter plasmid together with JDP2 and ATF3 expression plasmids into HEK-293 cells, and determined luciferase activity (Fig. 6e). Both JDP2 and ATF3 efficiently suppressed SDF-1 promoter to similar extent. In addition, the suppression of SDF-1 transcription was significantly higher in the presence of both JDP2 and ATF3 (Fig. 6e). These results further suggest that ATF3 and JDP2 directly regulate the expression of SDF-1, and therefore their double deficiencies, specifically in fibroblasts, contribute to SDF-1-dependent tumor growth.

## Discussion

Host cells within the tumor microenvironment are known to contribute to several hallmarks of cancer. Studies demonstrated that endothelial cells, fibroblasts and mesenchymal stem cells can promote tumor growth by different means including the induction of angiogenesis [31], contributing to tumor cell dissemination from the primary tumor [27], and immune system suppression [32], thereby allowing tumor growth and spread. Thus, dissecting the specific genes controlling this unique cross-talk between host and tumor cells within the primary tumor site is essential in order to identify new targets for cancer. In this study, we focused on the bZIP repressors, JDP2 and ATF3, which have been shown to associate with worse outcomes in patients [22]. While the tumor compromises both cancer cells and stroma, here we studied the role of ATF3 and JDP2 in the stroma. We found that tumors developed in mice deficient for both ATF3 and JDP2 grew faster than tumors grown in WT control mice. These effects were associated with changes in cell proliferation and blood vessel perfusion. Studies have demonstrated that increased angiogenesis is a hallmark of cancer [33], and thus could explain the increased proliferation rate of cells within the perfused tumor microenvironment. It was therefore important to delineate whether ATF3 and JDP2 expression is detrimental in the pro-tumorigenic microenvironment. We focused on the three most abundant stromal components in the majority of tumors, i.e., BMDCs, endothelial cells and fibroblasts. We asked whether each one of these components contributes to tumor growth in the absence of ATF3 and JDP2 expression. Fibroblasts were the major stromal component responsible for the increased tumor growth, since co-implantation of cancer cells with MEFs lacking both ATF3 and JDP2 was able to mimic the tumor growth phenotype developed in dKO mice.

We next hypothesized that the crosstalk between cancer cells and fibroblasts is mediated by a secreted factor regulated by the bZIP repressors. SDF-1 was identified as a putative factor that may account for the tumor growth characteristics. SDF-1 is associated with tumor growth and metastasis [34]. Its main receptor CXCR4 is expressed by circulating cancer stem cells of pancreas adenocarcinoma, which in turn contribute to metastasis [35]. In addition, SDF-1 is a potent chemokine that induces the recruitment of bone marrow derived cells to tumors, leading to increased angiogenesis and subsequent tumor growth [36]. SDF-1 specifically was shown to be secreted from myofibroblasts and affect epithelial cell proliferation and migration [37]. We recently demonstrated that SDF-1 is significantly upregulated in the circulation of mice treated with chemotherapy [38]. As a result, bone marrow derived endothelial precursor cells are highly mobilized, and subsequently home to the treated tumor site, leading to increased angiogenesis and tumor re-growth [38]. These effects are mediated by SDF-1, and its blockade, in combination with chemotherapy, inhibits angiogenesis and enhances the efficacy of chemotherapy. However, the relevant cells which express SDF-1 within the tumor microenvironment have not been tested in this study [38]. Here we show that SDF-1 levels are higher in serum derived from tumor-bearing dKO mice as compared to WT mice. Importantly, dKO MEFs secrete higher levels of SDF-1 compared to WT MEFs. The higher SDF-1 levels in dKO mice may explain the larger tumor size due to increased blood vessel perfusion. Notably, the secretion of SDF-1 was potentiated when cancer cells were co-cultured with MEFs. While the basal SDF-1 levels are transcriptionally regulated by ATF3 and JDP2, the potentiation of SDF-1 secretion following co-culture with cancer cells is most likely controlled post-transcriptionally. SDF-1 transcription is regulated by ATF3 and JDP2, and is probably not the sole protein expressed by fibroblasts. Other factors such as tumor growth factor β (TGF-β), interleukin 6 (IL-6) and various metalloproteases, are also secreted at the tumor microenvironment to promote tumor growth and metastases [39]. Further molecular studies are required to resolve the mechanism by which cancer cells manipulate fibroblasts to secrete pro-tumorigenic factors.

Previous studies have demonstrated that a single loss of either ATF3 or JDP2 in the stroma compartment resulted in reduced metastasis [23, 24]. We therefore initially hypothesized that tumor metastasis will be significantly abrogated in mice lacking both ATF3 and JDP2, as both transcription factors compensate for the loss of each other [25]. Surprisingly, while we observed an increase in tumor growth and augmented blood vessel perfusion, the number of metastatic foci in the lungs in any of the tumor types tested did not significantly change between WT and dKO mice. This raised the question for why the double deficiency of the bZIP repressors resulted in opposite metastasis phenotype, other than in a single loss of bZIP repressors. Metastases development is a multistep process, in which tumor cells disseminate from the primary tumor site, survive the circulation, and colonize at distant sites, in which future metastases appear [40]. We first suggest that the lack of ATF3 and JDP2 interferes with the loss of homeostasis among other bZIP family members. This suggests that the delicate balance between various members of the bZIP family is rather complex and not related only to ATF3 and JDP2. As such, the consequence on tumor fate cannot be predicted based on the results obtained from the deletion of each gene separately, especially in view of the fact that the expression of each of them compensates for the loss of its counterpart [25]. Recent studies demonstrated that tumor cells at the primary tumor site help promote a pre-metastatic niche, and therefore contribute to metastasis spread [41]. The colonization of host myeloid cells among other cell types within the pre-metastatic sites have been investigated, and SDF-1 is one of the factors associated with this process [42]. We found that fibroblasts expressing SDF-1 were sufficient to promote tumor growth and blood vessel perfusion. Evidently, SDF-1 knockdown in dKO MEFs resulted in significantly lower tumor size. In our dKO model we expected to detect less metastasis in the lungs, assuming there is a cumulative effect of each KO. However, the dKO model elevated the levels of SDF-1 in the serum and increased functionality of blood vessels, two parameters that previously shown to increase lung metastasis [28]. Therefore, it is possible that these opposite effects counteract each other, forming a similar level of metastasis in the dKO mice as detected in WT mice.

Lastly, it is possible that the double deficiency of ATF3 and JDP2 did not alter the metastatic process specifically in the experimental set-up of this study. We cannot exclude the possibility that metastasis would differ in different experimental conditions; for example, when dissecting the primary tumor and allowing for a more prolonged metastatic development as previously described [22, 23].

Additionaly, we addressed the question of how SDF-1 is regulated by ATF3 and JDP2. We demonstrated that the bZIP repressors are able to repress the SDF-1 promoter in HEK-293 cells and are likely to suppress SDF-1 expression in vivo. In contrast, another study showed that ATF3 activates SDF-1 transcription in N-Ras transformed epithelial cells, the HT-1080 cell line [30]. ATF3 and JDP2 can either activate or repress transcription depending on protein partner composition [12] and promoter context. Cell line difference and the different oncogenic drivers may explain this discrepancy. Nevertheless, in our study, we focused on examining the double deficiencies of ATF3 and JDP2. We assume that the effect on the SDF-1 promoter due to loss of expression can be further potentiated by other members of the AP-1 superfamily. Multiple AP-1 family members are able to positively regulate the SDF-1 expression via the association with the same TRE and CRE elements found within the SDF-1 promoter.

Collectively, our results suggest that the expression of ATF3 and JDP2 in stromal cells, and specifically fibroblasts, can regulate tumor growth. Therefore, identifying molecules that increase the expression of ATF3 and JDP2 can be used to control tumor growth and metastasis spread.

## Materials and methods

### Animals

This study was performed on age-matched WT (ATF3^+/+^; JDP2^+/+^), ATF3-KO (ATF3^−/−^; JDP2^+/+^) [43], JDP2-KO (ATF3^+/+^; JDP2^−/−^) [44] and dKO (ATF3^−/−^; JDP2^−/−^) female mice (10-12 weeks old). C57B/6 is the background for all mouse genotypes. All mice were bred and raised at the Pre - Clinical Research Authority (PCRA) at the Ruth and Bruce Rappaport Faculty of Medicine. All experiments were performed following the approval of the Ethics Committee for Animal Experiments at the Technion and according to the Guide for the Care and Use of Laboratory Animals of the National Institute of Health. All procedures were carried out under isoflurane anesthesia. MEFs were isolated from 15 d.p.c embryos as was previously described [16].

### Cell culture

Lewis lung carcinoma (LLC) and HEK-293 cell lines were purchased from the American Type Culture Collection (ATCC). PyMT murine breast carcinoma cell line was derived from primary tumor bearing transgenic mice expressing PyMT under the control of the murine mammary tumor virus promoter [23, 24] (kindly provided by Prof. Tsonwin Hai, Ohio State University). MEFs were immortalized using SV40 large T antigen. All cells were cultured in Dulbecco’s Modified Eagle’s Medium (DMEM) supplemented with 10% (v/v) FBS, 1% streptomycin and penicillin, 1% L-glutamine and 1% sodium pyruvate (full medium) at 37° C in a humidified atmosphere containing 5% CO_2_. Cells were tested routinely for mycoplasma. All cells were used within 4 months of resuscitation. All cell lines were tested and found to be free of mycoplasma contamination.

### Animal tumor models

LLC and PyMT cancer cells (0.5 × 10^6^ or 10^5^, respectively in serum-free DMEM) were subcutaneously implanted into the flanks and orthotopically implanted to the mammary fat pad, respectively, as previously described [23, 24]. For the LLC-MEFs co-implantation models, mice were either subcutaneously implanted into the flanks or into the mammary fat pad with Matrigel containing cell mixture composed of LLC cells (0.5 × 10^6^) together with MEFs (1.5 × 10^6^) and PyMT cells (10^5^) together with MEFs (3 × 10^5^), respectively. Tumor volume was monitored over time using the formula width^2^ × length × 0.5. Mice were sacrificed when tumors reached endpoint, or as otherwise indicated in the text. Perfusion assay was performed by i.v. injection of fluorescent DNA binding dye, Hoechst (40 mg/kg; Sigma-Aldrich, Israel), 1 min prior to mouse sacrifice as previously described [45].

### Bone marrow transplantation

Bone marrow cells were collected by flushing femurs and tibias from 7–8 week old WT or dKO mice. The BMDCs (1 × 10^7^ per recipient mouse) were transplanted by intravenous injection into lethally irradiated C57Bl/6 recipients. Irradiation was performed at 1000 cGy total body irradiation (250 cGy/min) using Elekta Precise (ElektaOncology Systems) linear accelerator 6 MeV photon beam radiation (Department of Radiation Therapy, Rambam Medical center, Haifa, Israel), as previously described [38]. Validation of bone marrow cell reconstitution was performed by PCR of genomic DNA obtained from mouse blood as previously described [38].

### Blood serum

Blood was obtained from the facial vein using 4 μm sterile Goldenrod Animal Lancet (MEDIpoint, Inc., Mineola, NY, USA). Blood was collected and allowed to clot for 2 h at room temperature followed by centrifugation of 15 min at 2000*g*. Serum was immediately aliquot and stored at −20 °C for further use.

### Conditioned medium assay

LLC, MEF or LLC and MEF (in the ratio of 1:3, respectively) cells were seeded in full medium at a concentration of 10^6^ cells/ml for 2 h. After the cells were attached to the plate, medium was replaced with serum-free medium and cultured for 24 h at 37 °C in a humidified atmosphere containing 5% CO_2_. Medium was collected and centrifuged to remove cells and cell debris. Samples were stored at −20 °C for further use.

### RNA Extraction and RT-qPCR

Total RNA was extracted from LLC and MEFs using an Aurum total RNA fatty or fibrous tissue kit (#732-6830, Bio-Rad, Hercules, California) according to the manufacturer’s instructions. Next, cDNA was synthesized from 1000 ng of purified mRNA using iScript™ cDNA Synthesis Kit (#170-8891, Bio-Rad, Hercules, California). Real-time PCR was performed using Rotor-Gene 6000^TM^ equipment (Bosch Institute, Sydney, Australia) with absolute blue SYBER green ROX mix (Thermo Scientific AB-4162/B). Serial dilutions of a standard sample were included for each gene to generate a standard curve. Values were normalized to heat shock protein 90 (HSP90) expression levels. The following primers were used:

ATF3-F GAGGATTTTGCTAACCTGACACC

ATF3-R TTGACGGTAACTGACTCCAGC

JDP2-F AGCTGAAATACGCTGACATCC

JDP2-R CTCACTCTTCACGGGTTGGG

SDF-1-F TGCATCAGTGACGGTAAACCA

SDF-1-R CACAGTTTGGAGTGTTGAGGAT

### Enzyme-linked immunosorbent assay (ELISA)

Quantification of SDF-1 protein levels in serum or conditioned medium (CM) was performed using a Solid Phase Sandwich ELISA kit (Mouse CXCL12/SDF-1 DuoSet ELISA, R&D Systems Inc. Minneapolis, Minnesota) according to the manufacturer’s instructions.

### Flow cytometry

Tumors harvested from mice were prepared as single-cell suspensions as previously described [46], cell suspensions were assessed for leukocytes (CD45), endothelial cells (CD31) and cancer associated fibroblasts (αSMA and CD140α) using flow cytometry, as described previously [47]. Flow cytometry experiments were performed using BD LSRFortessa™ (BD Bioscience). At least 100,000 events per sample were acquired.

### Immunofluorescence staining

Tumor sections were fixed in 4% formaldehyde overnight, embedded in OCT, and subsequently serially sectioned at 10 μm intervals. Frozen tumor sections were immunostained for CD31, Ki67 and counterstained with DAPI as previously described [45, 48]. Images were acquired using 3DHistech Pannoramic 250 Flash III (3DHISTECH Ltd., Budapest, Hungary). Each section was fully scanned, and for each analysis, five fields were randomly chosen and blindly and automatically analyzed using Image J software [49]. For every dot plot of image analysis, each dot represents the mean of the values taken from five fields, derived from a single mouse. Threshold was chosen for the identification of single nuclei (Hoechst) and dividing cells (Ki67).

### Antibodies

The following Antibodies were used:

CD45—Alexa Fluor® 700 (BioLegend, Catalog Number: 103128)

CD31—APC (BD Biosciences, Catalog Number: 551262)

αSMA—Actin, α-Smooth Muscle- FITC (Sigma Aldrich, Catalog Number: F3777)

CD140α—APC (BioLegend, Catalog Number: 135908)

CD31—Purified Rat Anti-Mouse CD31 (BD Biosciences, Catalog Number: 553370)

Ki67—(SP6) Rabbit Monoclonal Antibody (Abcam, Catalog Number: ab16667)

### Luciferase reporter assay

HEK-293T cells were plated at a density of 1 × 10^6^ cells per well in a 10 cm plate for 24 h prior to transfection. Cells were then co-transfected with 2 µg per plate of reporter plasmid pGL3, containing human SDF-1 promoter, upstream of the luciferase gene (kindly provided by Prof. Varda Rotter form the Weizmann Institute of Science) [30] and 100 ng of pCEFL-HA-ATF3 and JDP2 expression vectors. DNA content was adjusted to 10 µg with pCEFL expression vector. Cells were harvested 24 h post transfection. Subsequently, cell pellet was re-suspended in Promega lysis buffer. Cell lysate was tested for luciferase activity using the luciferase assay kit (Luciferase Reporter Assay System, Promega, CA, USA) and TD 20/20 luminometer (Turner Designs, Sunnyvale, CA, USA).

### Lentiviral transduction and shRNA transfections

For stable knockdown of SDF-1 in MEFs, lentiviral shHIF1A purchased from MISSION shRNA specific for SDF-1 was used (TRCN0000184347, Sigma-Aldrich, Israel), while the Control shRNA Plasmid-A (SC-108060, Santa Cruz Biotechnology, Inc., Dallas, Texas) was used as control. HEK-293T cells were co-transfected with shSDF-1 or shControl-A plasmids. Lentiviruses-containing medium from HEK-293T transfected cells was used directly to infect MEFs. Infected cells were selected with puromycin (2 µg/ml) for two weeks.

### Statistical analysis

Data are presented as mean ± SD. All mice were included in each statistical analysis, unless they were scarified at humane end-point before the end of the experiment. The animal genotype and experimental groups were blinded to the experimentalists during the experiment and data collection. Animals were selected for each group in a randomized fashion. Statistical significance of tumor volume was determined by Two-way repeated-measures ANOVA followed by Bonferroni post-test. The comparison between several means was analyzed by one-way ANOVA followed by Tukey’s post-test. Comparison between two means was performed by two-tailed Student’s *t*-test. All statistical analyses were performed using GraphPad Prism 7 software (La Jolla, California). *p* value < 0.05 was accepted as statistically significant. *p < 0.05; **p < 0.01; ***p < 0.001.

## Supplementary information

Supplemental data

Supplementary figure legends
