# Supplementary figures and images for: ATF3 and JDP2 deficiency in cancer associated fibroblasts promotes tumor growth via SDF-1 transcription
Source: Oncogene. 2019 Jan 22;38(20):3812–23. doi: 10.1038/s41388-019-0692-y (PMC6756089; doi:10.1038/s41388-019-0692-y)

S1

a

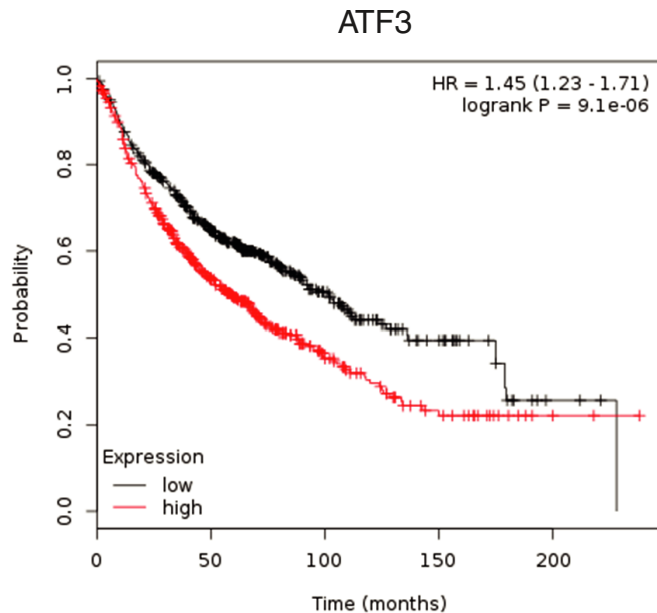

b

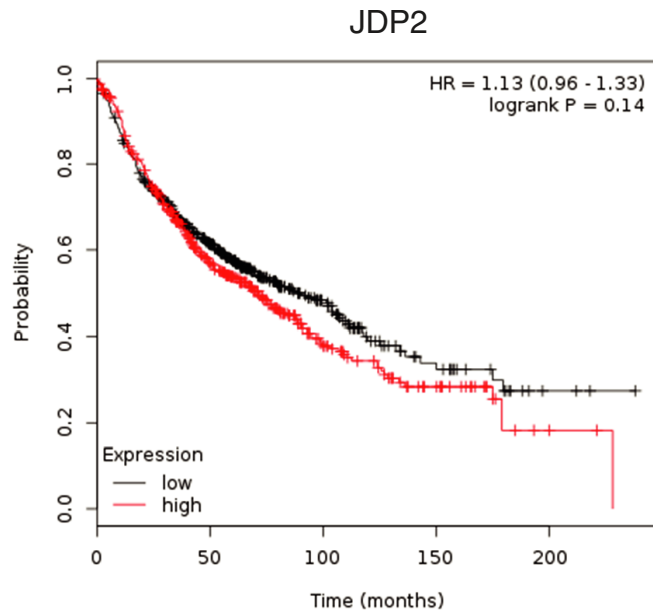

S2

a

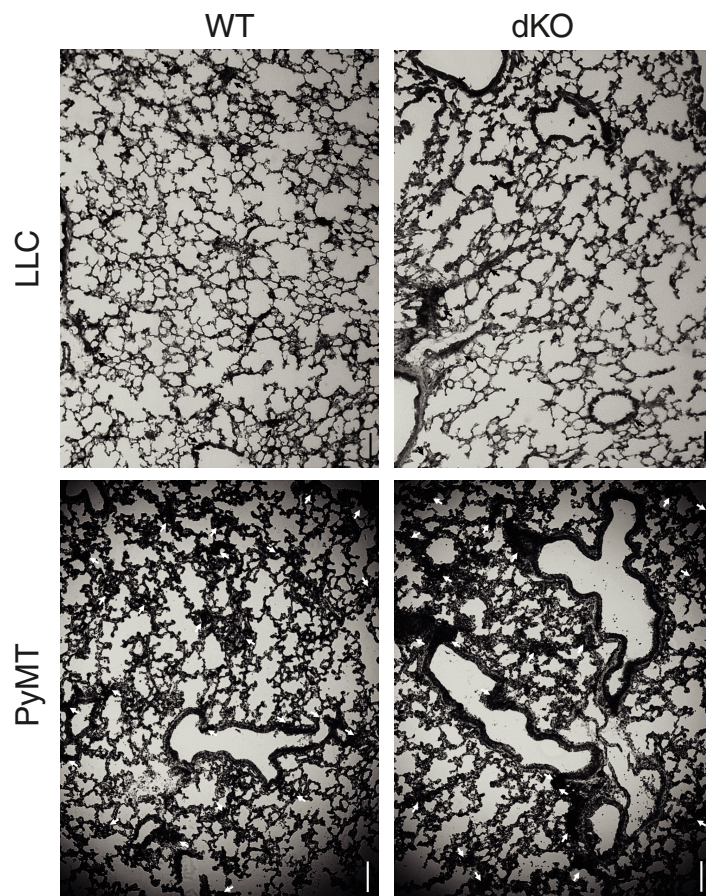

b

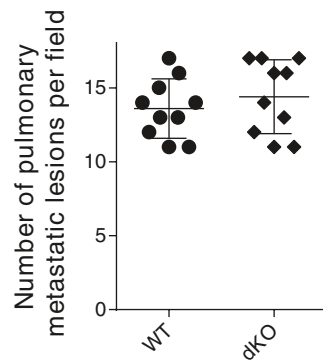

c

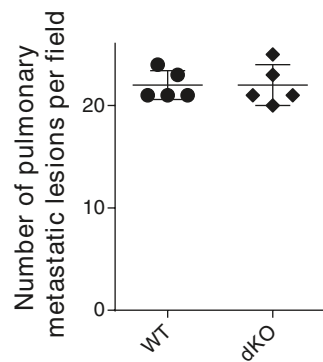

S3

a

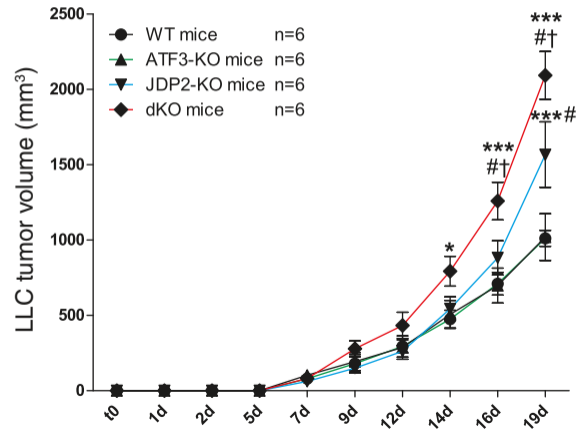

b

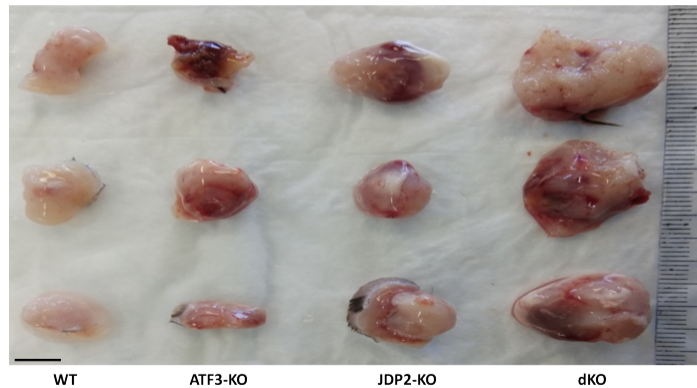

S4

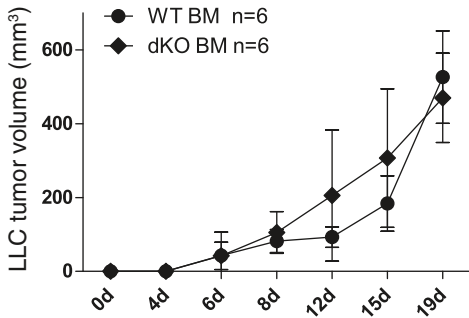

S5

a

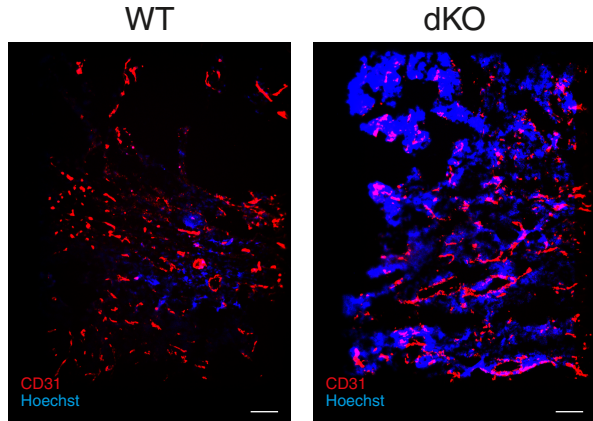

b

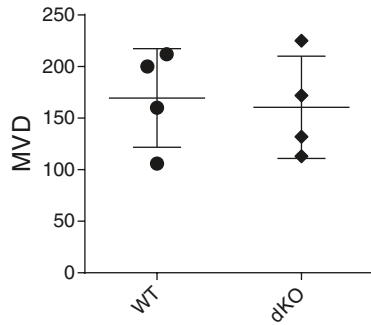

c

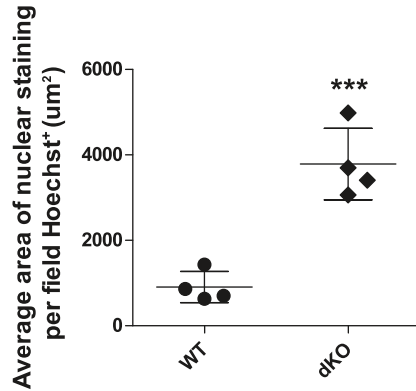

S6

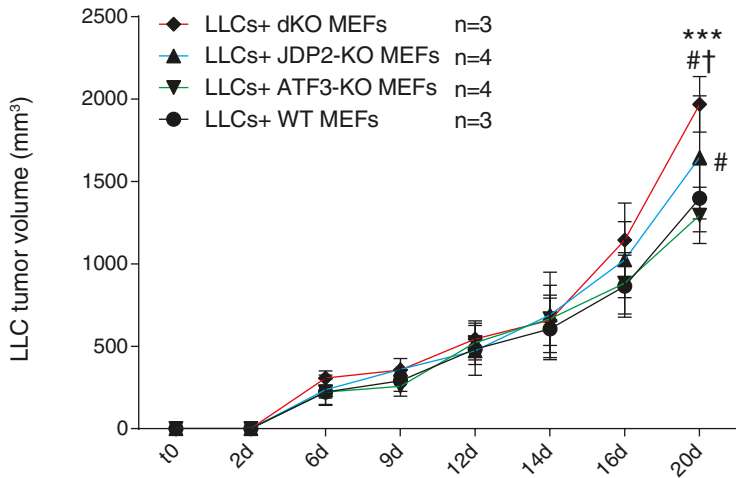

S7

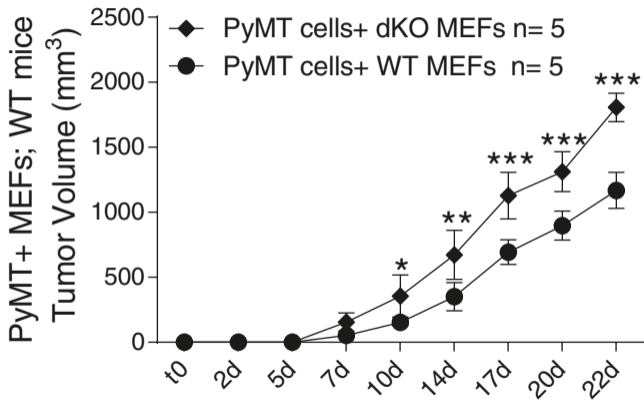

S8

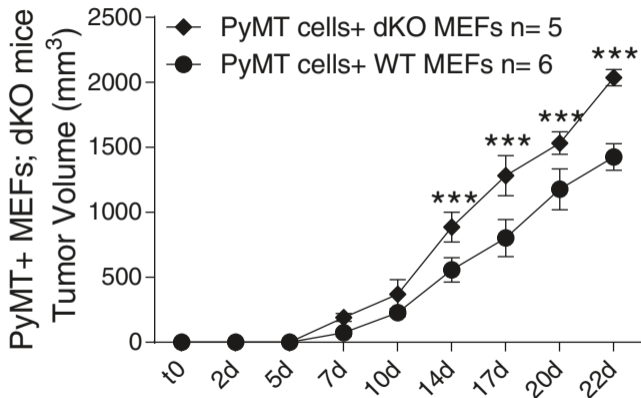



S10

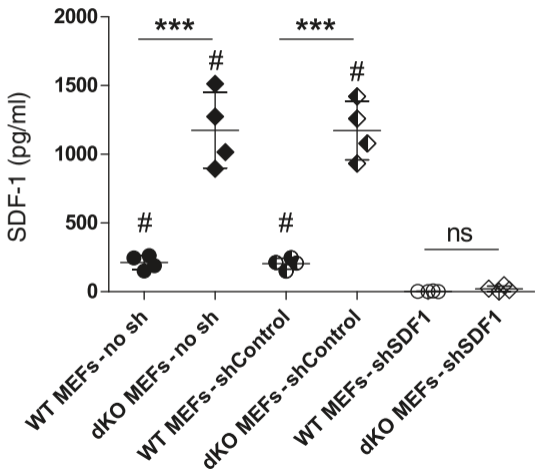

Supplement: Supplementary file 1 — Supplemental data [file 41388_2019_692_MOESM1_ESM.pdf]
