# Supplementary material for: ATF3 and JDP2 deficiency in cancer associated fibroblasts promotes tumor growth via SDF-1 transcription
Source: Oncogene. 2019 Jan 22;38(20):3812–23. doi: 10.1038/s41388-019-0692-y (PMC6756089; doi:10.1038/s41388-019-0692-y)
Supplement: Supplementary file 2 — Supplementary figure legends [file 41388_2019_692_MOESM2_ESM.docx]

**Supplementary Figure Legends**

**Figure S1**. **Kaplan Meier survival curves of lung cancer patients exhibiting high and low levels of ATF3 and JDP2.** Kaplan-Meier survival curves generated for ATF3 (**a**) and JDP2 (**b**) mRNA expression in 1,145 lung cancer patients based on Kaplan- Meier Plotter [http://kmplot.com/analysis/]. Expression above the median (red) or below the median (black).

**Figure S2**. **The number of metastatic lesions in the lungs of WT and dKO mice.** (**a, b**) WT and dKO mice were either subcutaneously implanted into the flanks with LLC cells (0.5x10^6^ cells per mouse) or orthotopically implanted into the mammary fat pad with PyMT cells (1x10^5^ cells per mouse). (**a**) Representative images of lung sections stained with H&E. White arrows indicate micro-metastatic lesions. Scale bar, 200 µm. (**b, c**) Quantifications of the number of pulmonary metastatic lesions per field in LLC (**b**; n=10) and PyMT (**c**; n=5). Each dot represents the mean of 5 fields taken from one mouse. Data are presented as mean ± SD, Student’s t-test, p>0.05. Representative image of at least two experiments.

**Figure S3**. **LLC tumor growth developed in various mouse genotypes.** (**a**) WT (black), ATF3-KO (green), JDP2-KO (light blue) and dKO (red) mice were subcutaneously implanted into the flanks with LLC cells (0.5x10^6^ cells per mouse). Tumor volume was monitored over time using the formula: width^2^×length×0.5. Data are presented as mean ± SD, two-way repeated-measures ANOVA followed by Bonferroni post-tests, n=6; * - difference compared to WT *p<0.05; ***p<0.001; # - difference compared to ATF3-KO; † - difference compared to JDP2-KO. (**b**) Representative images of tumors developed in various mouse genotypes. Representative image of at least two experiments.

**Figure S4**. **Chimeric mice harboring dKO BMDCs exhibit no difference in tumor growth**. Lethally irradiated (1000 rad) WT mice were transplanted with bone marrow cells derived from either WT or dKO mice. The chimeric mice were then subcutaneously implanted with LLC cells (0.5x10^6^ cells per mouse). Tumor volume was monitored over time. Data are presented as mean ± SD, Two-way repeated-measures ANOVA followed by Bonferroni post-tests, n=6, p>0.05.

**Figure S5**. **Tumors developed in dKO mice display higher blood vessel perfusion**. (**a**) Representative images of PyMT tumor sections derived from WT and dKO mice, stained with anti-CD31 (endothelial cells; red) and Hoechst (nuclei; blue). Scale bar, 100 µm. (**b**) Quantification of microvessel density (MVD) and blood vessel perfusion (**c**) per field. Each dot represents the mean of 5 fields taken from one mouse. Data are presented as mean ± SD, Student’s t-test, n=4, ***p<0.001. Representative image of at least two experiments.

**Figure S6**. **dKO MEFs promote LLC tumor growth**. WT mice were subcutaneously implanted into the flanks with Matrigel containing cell mixture composed of LLC cells (0.5x10^6^ cells per mouse) together with MEFs (1.5x10^6^ cells per mouse) derived from either WT (black), ATF3-KO (green), JDP2-KO (light blue) or dKO (red) mice. Tumor volume was monitored over time. Data are presented as mean ± SD, Two-way repeated-measures ANOVA with Bonferroni post-tests, n=3,4,4,3; * - difference compared to WT ***p<0.001; # - difference compared to ATF3-KO; † - difference compared to JDP2-KO. Representative image of at least three experiments.

**Figure S7**. **dKO MEFs enhance PyMT tumor growth in dKO mice**. WT mice were orthotopically implanted into the mammary fat pad with Matrigel containing cell mixture composed of PyMT cells (1x10^5^ cells per mouse) together with MEFs (3x10^5^ cells per mouse) derived from either WT or dKO mice. Tumor volume was monitored over time. Data are presented as mean ± SD, Two-way repeated-measures ANOVA with Bonferroni post-tests, n=5, * p<0.05; **p<0.01; ***p<0.001. Representative image of at least two experiments.

**Figure S8**. **dKO MEFs enhances PyMT tumor growth in dKO mice**. dKO mice were orthotopically implanted into the mammary fat pad as described in S7. Tumor volume was monitored over time. Data are presented as mean ± SD, Two-way repeated-measures ANOVA with Bonferroni post-tests, n=5, 6, ***p<0.001.

**Figure S9**. **SDF-1 secretion by MEFs is potentiated by the presence of cancer cells.** WT, ATF3-KO, JDP2-KO or dKO MEFs (7.5x10^5^) were co-cultured with LLC cells (2.5x10^5^). Cells were cultured in SF medium for 24 hours. The levels of SDF-1 in the conditioned medium were measured (pg/ml) by ELISA. Each dot represents one plate. Data are presented as mean ± SD, One-way ANOVA followed by Tukey's post-tests, n=4; * - difference compared to WT ***p<0.001; # - difference compared to LLCs. Representative image of at least two experiments.

**Figure S10**. **Levels of SDF-1 in shSDF-1 MEFs**. Conditioned media (CM) of untreated, shControl and shSDF-1 MEFs were collected after 24 hours in SF cultured medium. Protein levels of SDF-1 were measured by ELISA. Each dot represents one plate. Data are presented as mean ± SD, One-way ANOVA followed by Bonferroni post-tests, n=4; * - difference between genotypes, ***p<0.001; # - difference compared to SDF-1 knockdown (shSDF-1).
